# Restoration in Its Natural Context: How Ecological Momentary Assessment Can Advance Restoration Research

**DOI:** 10.3390/ijerph13040420

**Published:** 2016-04-13

**Authors:** Femke Beute, Yvonne de Kort, Wijnand IJsselsteijn

**Affiliations:** Human Technology Interaction, School of Innovation Sciences, Eindhoven University of Technology, P.O. Box 513, Eindhoven 5600, The Netherlands; Y.A.W.d.Kort@tue.nl (Y.K.); W.A.IJsselsteijn@tue.nl (W.I.)

**Keywords:** restoration, nature, experience sampling, quantified self, mHealth

## Abstract

More and more people use self-tracking technologies to track their psychological states, physiology, and behaviors to gain a better understanding of themselves or to achieve a certain goal. Ecological Momentary Assessment (EMA) also offers an excellent opportunity for restorative environments research, which examines how our physical environment (especially nature) can positively influence health and wellbeing. It enables investigating restorative health effects in everyday life, providing not only high ecological validity but also opportunities to study in more detail the dynamic processes playing out over time on recovery, thereby bridging the gap between laboratory (*i.e.*, short-term effects) and epidemiological (long-term effects) research. We have identified four main areas in which self-tracking could help advance restoration research: (1) capturing a rich set of environment types and restorative characteristics; (2) distinguishing intra-individual from inter-individual effects; (3) bridging the gap between laboratory and epidemiological research; and (4) advancing theoretical insights by measuring a more broad range of effects in everyday life. This paper briefly introduces restorative environments research, then reviews the state of the art of self-tracking technologies and methodologies, discusses how these can be implemented to advance restoration research, and presents some examples of pioneering work in this area.

## 1. Introduction

Many of our daily actions and interactions in the world pose adaptational demands. As a result, we may experience lower mood, increased physiological arousal, tension, or a decreased capacity for direct attention [1]. Restorative environments refer to physical surroundings or settings that produce beneficial effects by facilitating recovery from such demands (see for instance [2]). The majority of restoration research has looked at the recovery potential of natural scenery, although other environments—such as museums [3]—or environmental characteristics such as daylight [4] have also been suggested to hold restorative potential. See [4,5] for an extensive overview of restoration research. Mainly by contrasting natural environments with their built counterpart (urban scenery), researchers have demonstrated the benefits of our natural habitat on a number of indices relevant to human health and functioning, including lower stress levels, improvements in cognitive performance, and better mental and physical health (for an overview, see [4]). Evidence for these beneficial—or salutogenic—effects of nature have been found in controlled laboratory studies, field studies, cross-sectional studies, and epidemiological studies (see, e.g., [4]), yet the underlying mechanisms are not yet fully understood.

Several mechanisms have been proposed in the literature. The two most prominent theories focus on different antecedent conditions—one centering on stress and negative affect as the most salient demand condition [6,7], the other on mental fatigue, particularly of directed attention and self-regulation [8,9,10]. Stress-reduction is attributed to evolutionary-based, pre-cognitive affective responses [6,7] whereas attentional recovery is postulated to derive from cognitive processes (e.g., fascination, see [8,9,10]). In addition, there is some divergence in the qualities of environmental scenes they consider key to the recovery process (e.g., complexity and coherence [8] *vs.* presence of elements with survival value such as water, shelter from weather, and other sources of danger [6]). In reality, multiple processes may be in play [2,11,12].

In laboratory studies, restorative effects are generally tested by showing images or videos of natural *vs.* urban scenery after mental fatigue or stress induction. Similarly, field studies have tested the beneficial effects of walking in natural as opposed to urban environments. These studies focus on *acute* beneficial effects. A much-heard criticism of these studies is that they typically rely on a very limited selection of sampled scenes and hence do not do justice to the rich variety of natural and urban surroundings in the real world, often even reducing this to a crude dichotomy of “nature” *vs.* “not nature”. Cross-sectional and epidemiological studies do take place in a variety of realistic settings. These often relate residential proximity to green areas with health or wellbeing outcomes (see, e.g., [13,14,15]). However, given the unit of analysis in these studies, these too cannot inform us of differential responses to specific environmental aspects or characteristics. Moreover, the timeframe of effects investigated in these studies often has a longitudinal character, focusing on the cumulative effects of long-term exposure to nature, which are relevant but do not necessarily shed light on the underlying mechanisms. The current evidence base for restorative effects thus consists mainly of short-term (minutes/hours) and long-term (years) effects with only limited insight into the differential effects of individual contextual elements, and only scarce knowledge on the day-to-day dynamic between restorative environments and health and wellbeing.

The current paper wants to make the case that ecological momentary assessment or context-informed experience sampling methodologies could aid in filling this gap between relatively short-lived *vs.* longitudinal effects, and between tightly defined yet limited samples of environments *vs.* high-level comparisons of environmental categories, especially since experiencing restorative environments is an inherent part of our daily lives. Therefore, studying the relation between environment and health and wellbeing in the realm of everyday life appears both timely and relevant.

## 2. Experience Sampling and Ecological Momentary Assessment

In the early 1970s, Csikszentmihalyi, Larson, and Prescott [16] introduced experience sampling as a way to measure human experience in the realm of everyday life. Experience sampling aims at capturing daily fluctuations in psychological constructs through the use of repeated diary entries. Recent developments in mobile technology have re-invigorated this research, lifting it from a paper-and-pencil-based methodology to context-aware measurement with seemingly endless opportunities. An important merit of measuring human affect, cognition, behavior, and physiology in everyday life is the ability to simultaneously capture contextual circumstances, thereby substantially fostering both ecological validity and the potential to study person-environment interactions. This holistic approach is especially relevant for restoration research, where the focus is on the beneficial effects of person-environment transactions on health and wellbeing.

Ambulatory assessment of emotions, physiology, behavior, and performance is not only of interest for researchers, but also increasingly taken up by consumers on their own initiative. Within this movement, also labeled the Quantified Self movement by Gary Wolf in 2007, there is a strong belief that tracking personal data aids in personal development towards, for instance, happiness or an improvement in cognitive or athletic ability. As a result, a wealth of apps is available to support self-tracking of data on a wide range of outcomes (for an overview, see: [17]). These apps often use sensors that are either embedded in mobile technology or can communicate with it. The availability and fast-paced uptake of these technologies offer far-reaching opportunities for researchers interested in diary research. The present article focuses on these developments and how they can be utilized to advance restoration research. Our purpose is not to present a comprehensive and systematic review. Rather, we will discuss the state of the art of self-tracking technologies and experience sampling methodology and focus on their utility for restoration research.

To find relevant articles, we conducted an extensive search on PubMed, Psychinfo, and Google Scholar. Keywords used in this search were: “ecological momentary assessment”, “ecological momentary intervention”, “experience sampling”, “quantified self”, “personal informatics”, “diary studies”, “smartphone”, “mHealth”, “context”, “context-sensitive”, “restoration”, “recovery”, “green”, “wilderness”, and “natural environments”. We also inspected relevant references cited in these articles. This yielded articles from very different research domains, including psychology, clinical studies, economics, and computer sciences.

This paper will start with an introduction to experience sampling methodology, after which we will introduce context-enriched measurements. The third section will focus on the current application of self-tracking in restoration research, after which we will discuss the most promising future research directions.

## 3. Experience Sampling Research

In this section we will briefly introduce the experience sampling methodology and related methods. For a more thorough introduction into the methodologies, please consult [18,19,20,21,22,23].

### 3.1. A Quick Guide to Experience Sampling Methodology

Tracking human experiences in everyday life is at the core of the Experience Sampling Method (ESM; [16]). In fact, a rich history of trying to capture daily life exists extending far beyond ESM, including for instance Brunswik’s [24] random sampling of situations (for a historical overview, see: [25]). In addition to self-reports of affect and experience, experience sampling may also incorporate ambulatory assessment of the context, behavior, or physiology such as cardiovascular monitoring [18]. As this sensor-enriched methodology now allows for chronicling far more than subjective reports alone, some people prefer the term ecological momentary assessment (EMA) over ESM. The term ESM is also more commonly used in psychology, whereas EMA is utilized more in clinical research. Others, including ourselves, use the terms interchangeably.

High ecological validity is one of the main merits of sampling daily life *in situ*. Laboratory studies enable maximum experimental control, but real life cannot always be captured in these controlled environments. Therefore, this high internal control sometimes compromises the generalizability of results to the real world (see, e.g., [19]). In addition, human behavior often is not the direct response to a single predictor factor. Rather, contingencies result from a complex interplay of individual, situational, and contextual cues and factors. Context-enriched measurement offers the chance to consider not only a wider selection of determinants of human behavior, but also a broader collection of outcome variables than is typically possible in the confines of a lab. Furthermore, it allows for a more detailed inspection of the complex interplay of human behavior and situational factors [20].

A second advantage that is frequently mentioned in comparing ESM to more traditional laboratory and survey studies is the reduction in retrospective bias as participants are no longer asked to indicate for instance how they *felt* but rather how they *feel at this moment*. Indeed, a number of studies have indicated that retrospective reports of experiences and cognitions are subject to distortion. Reasons for this distortion are, for instance, interference by current mood states, overrepresentation of salient events, or recall biases (see, e.g., [18,19,20,21,22]).

Besides high ecological validity and reduced retrospective bias, daily measurements have another powerful asset. They allow for investigating effects *within* individuals, rather than only *between* individuals. In other words, inferences can now be made on the individual level as well as the group level (see e.g., [24]). With the aid of advanced statistical analyses such as time-series analysis, temporal dynamics and variation of the data within a single individual are utilized to investigate lagged effects and even causation, in contrast to cross-sectional and epidemiological studies. In addition, these analyses allow inferences on the individual level rather than for the “average person”—who, arguably, does not exist [26].

Proponents of this individual-level approach have argued that conventional psychological research focuses too much on group-level outcomes. To illustrate this point, let us look at the relation between stress and psychosomatic complaints. There is a general belief that an increase in stress will lead to more psychosomatic complaints [27]. A recent study, however, investigated the relation between stress and psychosomatic complaints in individuals with functional somatic symptoms (*i.e.*, clinically unexplained symptoms) [27]. They found that for some individuals increased stress levels caused the deterioration of their psychosomatic complaints, whereas for others this relation was opposite, reversed, mixed, or even non-existent. Similarly, the (temporal) relation between environmental exposure and health may differ between individuals. This research shows that even though—in general—stress may lead to more psychosomatic complaints, the strength and even direction of the causal relation can differ substantially between individuals.

A (quasi-) experimental design can also be achieved by including an intervention in the sampling protocol. This subset of EMA is often referred to as Ecological Momentary Interventions (EMI; [28]) and is often applied within the clinical domain as part of mHealth (mobile health) interventions. These interventions aim at behavior change or improving disease management and aim for instance at smoking cessation, controlling hypertension, or reducing depressive symptoms (for an overview, see e.g., [29,30,31]).

### 3.2. The Experience Sampling Protocol

The Experience Sampling Method (ESM) essentially asks people to interrupt their activity at certain times throughout the day and report their experience in real time. A variety of technologies can be used to help participants record their activity, ranging from a stopwatch and a paper-and-pencil journal to mobile phones and smart watches for signaling as well as note-taking.

Different sampling strategies exist, the most important types being signal-contingent, event-contingent, and continuous (automatic) protocols. With signal-contingent sampling, participants receive a beep every time they need to fill in the questionnaire. These beeps can be distributed randomly or at fixed intervals. Participants may also be asked to fill in the questionnaire on their own initiative, whenever a certain event has occurred (event-contingent), such as having just smoked a cigarette or after every social interaction. With continuous protocols, contextual (e.g., light exposure) or bodily aspects (e.g., heart rate) are monitored 24/7 through ambulatory assessment. Each sampling strategy has its own advantages and disadvantages. For an overview, see [32].

The questionnaires used in ESM protocols often differ on a number of dimensions from the ones used in laboratory or survey studies. First of all, whereas survey studies often ask for answers aggregated over a certain time span (e.g., last week, or over the last three months), ESM questions often probe an individual’s current state or their behavior since the last beep. Second, given the high time burden of these studies and the fact that questions are answered multiple times per day, outcomes are often measured with a single item rather than a validated scale consisting of multiple items. In addition, as researchers are often looking for diurnal variations in outcomes, one should also pay attention to choosing the right items to capture these dynamic patterns. For instance, when measuring mood, it makes more sense to measure a frequently altering emotion—such as being sad—rather than probing for extreme (and usually more rare) emotions, such as feeling depressed.

### 3.3. Limitations of Experience Sampling

Filling in the same questionnaire multiple times per day can place a considerable burden on participants. This may trigger reactivity to the study protocol, meaning that participants alter their behavior and/or thoughts in response to the measurement protocol. One should always be alert to the occurrence of reactivity as it goes against the main premise of ESM: measuring everyday life as it is lived. Reactivity can also occur in response to the use of contextual sensors (e.g., when they are highly visible) or by sensitization to inner mental states or behaviors previously unknown to them.

Additionally, even with random sampling not all situations may be sampled [20,33]. Some situations may be more prone to participants dismissing beeps, such as while driving a car or when under great time pressure. In addition, rare events are likely to be missed even with frequent random sampling. See [20] for a more thorough discussion of the limitations of ESM.

To overcome some of the pitfalls of ESM (e.g., reactivity and drop-out due to time burdens) as well as to better capture context, traditional ESM protocols are now increasingly enriched with ambulatory measurements from mobile sensors.

## 4. Seemingly Endless Possibilities: Context-Enriched Sampling

Context-enriched measurement combines experience sampling with smart sensing of contextual, physiological, and behavioral factors including the physical environment, but also bodily responses, social components, or situational factors (e.g., being alone or in the presence of others). As sensors are becoming increasingly smaller and less expensive, the possibilities for context-enriched measurement increase. In fact, many smartphones now already incorporate numerous sensors such as GPS, gyroscopes (measuring rotation), and temperature and light sensors. In addition, the majority have a high-resolution camera, sound recording options, Bluetooth, and Internet connectivity. Each sensor on its own can provide the researcher with useful data on a specific target parameter, such as location or activity level. Combining multiple sensors can create synergetic outcomes, allowing for inference of the type of activity or emotion (e.g., coupling physiological parameters with voice intonation). These sensors need not all be smartphone-based. In fact, Bluetooth**^®^** and Internet connections allow ubiquitous computing: communication between multiple devices. Sensors can thus also be wearable (such as a heart rate strap around the chest, or a light logger clipped to the shirt), present in the (smart) environment, or even embedded in clothing.

In the present age of the Internet-of-Things, an astonishing range of devices has the ability to communicate with each other. For instance, weighing scales can connect to an app on your smartphone and Power-over-Ethernet lighting systems allow interactive and distant control over light settings as well as collecting data through sensors attached to the lighting system (see, e.g., [34]). Communication between devices could further signal proximity to other people, thereby presenting a measure for social company or level of crowding (e.g., via Bluetooth**^®^** communication). Lastly, relevant data could also be extracted from coupling data collection with media use (e.g., number of e-mails received) or with someone’s digital agenda. Obviously, some of these options sound more futuristic, will require a higher level of engineering, and are more intrusive in participants’ lives than others.

Candidates especially relevant for enriching experience-sampling studies pertain to the categorization of the environment (e.g., camera images, GPS), activity level (e.g., accelerometer), physiological measurements (e.g., heart rate), and ambient conditions (e.g., sound, light, air quality). In addition, passive telemetric monitoring could help infer type of activity and social interactions without explicitly probing participants for this information. Advanced algorithms could further automatically register emotional states from sensor data, such as speech or physiological measures.

### 4.1. Global Positioning System (GPS)

GPS information can be used to deduce not only current position, but also to create a time-location history, storing where people have been and for what duration. Coupling this information with a GIS database further allows for categorization of environments, for instance with relation to the amount of greenery in an individual’s direct surroundings. Location information could further be applied to create location-based triggers for sampling experiences at pre-determined places. The spatial resolution of GPS monitoring will still, however, not capture more subtle variations in environmental characteristics. For instance, it will not be able to distinguish between being in an office with a view *vs.* being in an office without a view to the outside. Therefore, additional contextual measurements could be necessary to establish an even richer set of restorative environments.

### 4.2. Video and Audio Recordings

Automatic tracking of audio and video data would allow for more detailed categorization. In life-logging applications, this feature is already integrated. Life-logging consists of a camera that a person wears and that takes images automatically at regular intervals, which—for instance—has been utilized to investigate travel behavior [35]. In addition, from recording sound snippets at regular intervals (see, e.g., [36]) a variety of parameters can be inferred such as the type of activity or social company. These techniques obviously require ethical consideration as the privacy of not only the participant, but also of uninformed bystanders is at stake. Oftentimes, these issues are dealt with by automatically processing the data on the device itself. For instance, algorithms developed within the emerging domain of Social Signal Processing [37,38] can be used to detect human emotions or characterize social events from speech, such as stress [39] or laughter [40], obviating the need to store full-blown microphone or video feeds on a device.

Collecting audio fragments and photos can be used not only to categorize environments, but also to capture more qualitative responses of participants, a protocol sometimes referred to as descriptive-experience sampling [41]. Audio and camera capabilities of smartphones can help collect rich qualitative data including verbal responses to questions, and video or sound bites from the environment deemed relevant by the participant. A nice example of the use of this more qualitative approach was provided in a recent study investigating the feasibility of these types of applications to better understand self-harming behavior [42]. Study participants were invited to keep a multimedia diary in which they could post videos or recorded text. In addition, they had access to a private blogging site dedicated to the research. The qualitative data collection was combined with ambulatory assessment of heart rate and activity level.

### 4.3. Actigraphy

Actigraphy enables 24/7 monitoring of duration, intensity, and frequency of movement [43] and has already been used in a wide range of research areas. The functionality of these sensors can go beyond mere activity level. For instance, they can also be used to measure sleep quality [44] and posture [43]. Combining actigraphy with GPS can help infer or disambiguate activity (for instance driving a car at high speed *vs.* walking).

### 4.4. Ambulatory Physiological Measurements

A fourth class of sensors highly relevant for restoration research consists of ambulatory physiological measurements. Again, these technologies allow capturing a wide variety of parameters, such as skin conductance, heart rate, heart rate variability, breathing rate, or even electroencephalography (EEG; for an overview, see: [45]). Combining ambulatory measurement of bodily processes with GPS allows for geo-referenced monitoring of physiological parameters, which could provide a continuous and rich dataset linking environment type with wellbeing.

### 4.5. Next-Generation ESM

Not only can technological advancements be used to enrich measurements, they may also be employed to improve the methodology itself. These “next-generation” ESM protocols are still in their infancy, but most aim at finding innovative and interactive protocols to facilitate long-term tracking in research protocols (e.g., aiming at lower drop-out and increased motivation). Context-sensitive measurement, for instance, [34] uses information gathered continuously to determine when questions should be asked, for instance when a person enters a specific area, is engaged in a certain activity, or when in a specific emotional state. This way, the time burden on participants will be substantially lower as beeps will only sound when necessary. Other strategies include gamification of the research protocol [46] or tailoring the protocol to the individual [47].

The opportunities presented for context-enriched measurements are legion, but they will require a multidisciplinary approach as collaboration with computer science will be required. Additionally, it will be a challenge to find the right combination of sensors to distill meaningful data as well as the right balance between the number of wearables and privacy issues and reactivity of the participants. Much will also depend on the reliability of the apps, sensing devices, and algorithms, which may not always be thoroughly validated. Setting up an experiment with smart sensing of context will probably be more time-consuming than creating a laboratory experiment. Programming for multiple platforms (e.g., both Android and iOS) and multiple sensors will require a substantial programming effort by an experienced app developer. However, if designed well, the dataset can potentially be richer than a traditional laboratory experiment.

## 5. The Quantified Self in Nature: Examples of Current Implementations in Restoration Research

A small number of studies have already pioneered the implementation of ESM in restoration research. This section will present some examples of these studies including experience sampling studies and research utilizing opportunities presented by ambulatory assessment. We have focused here on studies investigating the beneficial effects of nature exposure.

### 5.1. Paper-and-Pencil Experience Sampling Studies

One early example of ESM in restoration research studied the dynamic, emergent nature of on-site wilderness experiences. The study employed on-site pen-and-paper surveys presented at different times during a single wilderness experience (e.g., [48]). This study, for instance, indicated that experiences of focus and connectedness changed over the course of a wilderness visit [48]. A structured paper diary was implemented in a second study focusing on the restorative qualities of favorite places, revealing that prescribing participants a visit to favorite places on a daily basis can be effective in increasing restorative outcomes [49].

In a third study [50], paper-and-pencil diaries were combined with pagers to explore the vitalizing effects of being outdoors and in nature in daily life. The “naturalness” of the environment was measured using a semi-objective checklist, consisting of a number of typical natural and urban objects. Overall naturalness scores for every sampled environment were computed by counting the number of natural elements checked on the list and subtracting the number of urban elements. The checklist thus offered a low-tech yet still user-friendly means of categorizing settings on a scale of urban–natural, rather than imposing a dichotomous distribution. The study yielded higher vitality scores for participants in environments with an increasing number of natural elements.

The majority of these studies were still relatively “low-tech”, using traditional paper-and-pencil methods, with the main disadvantage being a lack of control over whether participants actually fill in the questionnaire shortly after the beep. However, some studies have also used experience sampling protocols offered on digital media. Other studies have exploited the opportunities provided by ambulatory sensing technology in field research.

### 5.2. Digital Experience Sampling Protocols and Ambulatory Assessment of Context

In one pilot study, researchers employed the same semi-objective environmental checklist to measure the amount of nature in the environment as mentioned above [50], but implemented this on a smartphone in an experience sampling protocol that lasted six days. A very similar semi-objective checklist was designed to also estimate the amount of daylight at the participant’s location. The study revealed promising effects of naturalness and daylight on both mood and preference [51].

A second study used a personal digital assistant (PDA) based experience sampling study and investigated the efficacy of a nature education program as compared to a passive leisure task for children [52]. The researchers learned that the outdoor activities significantly improved mood, but yielded lower scores on flow and challenge than the regular leisure activities of the children.

On a much larger scale, MacKerron and Mourato [53] launched a commercially available app (Mappiness) combining GPS with happiness ratings for a period of six months. The researchers gave participants control over the frequency and timing of the experience sampling protocol. During this timeframe, they were able to collect responses from more than 20,000 participants (a total of more than a million responses) and used this data to confirm that happiness is indeed higher in natural environments. In their study, they exploited the increasing tendency of people to track their own behavior and thoughts. Instead of only distributing the experience sampling tools among dedicated research participants, they opted to develop a commercially available app [53]. The disadvantages of this method are a loss in experimental control and an increase in self-selection bias.

Doherty, Lemieux, and Canally [54] also combined GPS tracking with ESM. They explored a method to track activity levels and wellbeing in natural environments and combined GPS tracking with experience sampling questions measuring momentary health and wellbeing. In addition, they added five open questions probing the experiential aspects of nature visits. Participants could verbally record their answers. As their study was only designed to test the method, they did not provide results of nature on health and wellbeing but they did conclude that the method was viable and yielded both quantitative and rich qualitative data (from the audio recordings).

GPS tracking is also used in field studies to ensure that participants stick to pre-determined routes (e.g., [55]). The main advantage of this is that participants no longer have to walk the route chaperoned by the experiment leader. In addition, geo-referenced monitoring of physiological parameters can be used to couple GPS data with physiological responses. In one study, participants were instructed to walk a prescribed route that led them past a number of study areas (vacant lots) while their cardiovascular responses were monitored—directly coupled with the GPS coordinates [56]. By singling out these areas of interest in the data, they were able to establish the beneficial physiological effects of planting vegetation in these vacant lots.

Ambulatory physiological measurement has also been implemented in a number of field studies in the restoration domain. Mobile EEG measurement has been employed to establish the benefits of physical activity in green space (as opposed to city/commercial areas) [57] and brain activation when experiencing natural *vs.* urban scenery [58]. Both studies consisted of a field study with a duration no longer than 30 min, which already signals one of the potential difficulties to overcome in 24/7 monitoring: limitations in battery life and data capacity for some commercially available physiological devices.

Lastly, a number of studies have further investigated environmental influences on activity levels, which are relatively easy to track with off-the-shelf or even built-in accelerometers. These studies have demonstrated that children are more active in rural and green areas. Schoolchildren in rural environments have a higher daily activity level than children going to school in urban regions [59]. In addition, combining actigraphy with GPS tracking, Jones, Coomes, Griffin, and Van Sluijs [60] were able to point to the importance of green areas for the activity level of children.

The examples above indicate that studies are already utilizing ambulatory assessment and experience sampling methodologies. Many opportunities, however, have not yet been exploited to their full potential. The next section will point out a number of areas for which self-tracking provides a timely tool for advancing restoration research.

## 6. The Quantified Self in Nature: Future Implementations in Restoration Research

It is virtually impossible to review and discuss all current and future research directions for ESM/EMA in restoration. However, we have aimed at identifying the main areas in which self-tracking could help advance restoration research by exploiting the benefits of daily-life research: increased ecological validity, overcoming retrospective bias, and increased opportunities for studying intra-individual effects. These main areas are: (1) capturing a rich set of environment types and restorative characteristics; (2) intra-individual *vs.* inter-individual effects; (3) bridging the gap between laboratory and epidemiological research; and (4) advancing theoretical insights by measuring effects in everyday life. In the next section, we will first discuss each main area followed by a discussion of how self-tracking could help advance research in this area. We will end this section by looking at opportunities for (quasi-) experimental studies using experience sampling.

### 6.1. Capturing a Rich Set of Environment Types and Restorative Characteristics

#### 6.1.1. Rationale

As was briefly reflected on in the introduction, a frequently mentioned critique on restoration research is the strict dichotomy between natural and urban environments (see, e.g., [61,62]), especially in laboratory research, where nature is almost exclusively contrasted with urban scenes (see e.g., [4]). This strict separation between urban and natural environments does not reflect the natural variety in environments occurring in the real world and may also not be in line with how people generally judge environments [63]. In addition, even though much of the research within the field of restorative environments has focused on natural environments, restorative urban environments—often related to leisure—have been identified as exhibiting restorative potential as well (e.g., art galleries, [3]).

The rich—and natural—variation in the environments encountered in everyday life captured with experience sampling methodologies could help shed light on the exact environmental characteristics that cause an environment to be restorative. This more nuanced set of environment-health interactions could further help distinguish between health-detrimental effects of urban environments (e.g., noise and crowding) *vs.* salutogenic effects of nature, which represents another persistent issue within restoration research (see, e.g., [4]).

Ambulatory measurements can aid in increasing the spatial resolution of the environments encountered. The importance of place and context for health has been gaining traction within the public health domain over the last few decades (see, e.g., [64,65]), expressed in research domains such as medical geography. Similarly, a growing research effort has been geared towards finding relations between green spaces in the proximity of the home and health outcomes such as general health [13], mental health [14], and mortality [15].

Existing epidemiological studies within restoration research, however, often use (current) place of residence for studying the relationship between nature and health outcomes (e.g., [13,15]) whereas people often live and work in multiple places over a lifetime and encounter a large variety of environments on a day-to-day basis [65]. A more fine-grained spatial resolution of location information would significantly advance the existing knowledge base for place effects on health and wellbeing.

An additional advantage of ESM in people-place research is that it enables measuring both individual as well as contextual contingencies. Mitchell and Popham [15] postulate that the stress-reducing effects of nature explain the health benefits of more green space in the proximity. In response, Hartig [66] pointed to the interdependency of access to natural green areas and an individual’s amount of physical activity, which—on its own—has well-established health benefits (see e.g., [67]). It is exactly this interdependency between contextual effects (e.g., access to green areas) and individual characteristics (e.g., athletic fitness) that complicates the study of place and health by clouding the direction of causation [68]. In the case of the study presented above, does more nature in the proximity have health-protective effects on its own, or do people living in greener areas have better health because they are more able to engage in physical activity?

Besides providing a richer selection of potential restorative environments, context-enriched measurements will also enable capturing multi-modal experiences as well as other relevant contextual contingencies for human behavior and wellbeing. The prolonged parallel assessment of indicators of mood and health, on the one hand, and environmental features on the other, allows for an investigation of subtle person-environment transactions. For instance, in the present evidence base for restorative environments, the visual benefits of nature dominate, whereas other senses such as touch, smell, and sound could also contribute. In addition, nature exposure often coincides with other contextual factors that could also benefit health and wellbeing, such as social company [69] and daylight exposure [4]. Combining this rich set of contexts with frequent measurements of health and wellbeing will significantly aid us in understanding the full restorative potential of our physical environments.

#### 6.1.2. Self-Tracking Opportunities

On an elementary level of self-tracking, researchers could just ask participants to report where they are (see, e.g., [49]) or to describe the elements present in the environment (see, e.g., [50,51]) and link this with self-reports of wellbeing. Tracking environmental exposure could also be executed more automatically using contextual sensors (such as GPS, light loggers, microphones, or cameras). An advantage of these ambulatory measurements is that one acquires a continuous dataset of where a person has been, rather than only where they were at the time of a beep. In addition, one can measure the effects of the environment (e.g., amount of nature) on a variety of health determinants without sensitizing participants to the environment itself (thereby avoiding probing for “laymen’s theory” when the participant has guessed the purpose of the study).

GPS measurements not only provide information about where a person has been, but also on the timing and duration of this person-environment encounter. Combined with ambulatory measurements of activity and/or physiology, this could help build a higher-resolution dataset for analyzing place effects on health. An even more fine-grained dataset can be achieved using life-logging applications. These applications, combined with smart algorithms, could automatically detect, for instance, the amount of greenery in the direct environment. In addition, a wealth of environmental sensors is available to enrich the dataset with other contextual factors, including light levels, temperature, air quality, and sound levels.

### 6.2. Distinguishing Intra-Individual from Inter-Individual Effects

#### 6.2.1. Rationale

Self-tracking not only allows for higher spatial resolution, but also enables investigating temporal dynamics and relationships. Many aspects of human physiology, cognition, and behavior fluctuate throughout the day, for instance, under the influence of our biological clock (see e.g., [70,71]). These dynamics are difficult to capture in laboratory or survey studies, but can provide us with valuable insights. Similarly, both the need for restoration as well as the efficacy of restorative environments may fluctuate throughout the day. Diurnal patterns and dynamics in mood have already provided additional insights into the pathology of various mood disorders (see, e.g., [22,72]). The dynamics of mood in relation to contextual factors, such as sleep, physical activity, and negative events provides especially valuable lessons [73,74]. The naturalness, or restorative potential, of the environment is a suitable candidate for such a contextual factor. Furthermore, mental health is at the core of restoration research and psychiatric populations are given increasing attention in restoration research (e.g., [55,75,76]).

Knowledge of the daily fluctuations in wellbeing and need for restoration allows for a number of inferences. First of all, as mentioned earlier, it enables investigating temporal patterns, establishing when the need and efficacy are highest. Second, advanced statistical analyses could reveal temporal causality and will help distinguish between recovery and the buffering effects of restorative environments. More specifically, do beneficial effects only occur *after* depleting resources or stress induction (recovery) or could exposure to nature also help buffer against future negative events (see, e.g., [77])? In addition, it could provide information about the right “dosage” (*i.e.*, how long does a person need to be exposed to a restorative environment to experience benefits?), and the extinguishing time (how long before benefits wear off?) and individual differences in these aspects. Rather than only testing the effects of “forced” exposure to these environments, one could also inspect whether (some) people visit restorative environments on their own initiative with the purpose to recover, and under what circumstances.

Third, individual differences in restoration can be revealed, potentially leading to the development of different “restoration personality types”. Persistently, people question whether being in nature will aid everyone. In fact, as Pearson and Craig [61] correctly point out, the majority of the world population explicitly chooses to live in urban areas. By investigating the effects of the environment at the individual rather than general level, valuable lessons could be learned concerning the individual benefits of nature exposure; this could also allow testing to see whether the restorative effects of nature are indeed as universal as many preference studies lead us to believe (see e.g., [78,79]) or whether specific phenotypes exist for the benefits of nature. This knowledge is, for instance, especially relevant for the design of therapeutic interventions. ESM enables answering the question of whether there are individuals who sometimes thrive in the buzz of city life. Moreover, what separates them from “nature-lovers”? Under what circumstances does someone benefit from a restorative environment, and how does this differ between and within individuals?

#### 6.2.2. Self-Tracking Opportunities

To answer these questions, it is important to study the daily dynamics of restoration and the covariation of environment type and wellbeing, but also its lagged effects and intra-individual patterns. Experience sampling methodologies employing self-reporting measures appear especially valuable for gaining a better understanding into the daily dynamics of restorative environments and restorative outcomes. Preferably, these studies employ digital recording (to increase control over the timing of the answers) of these experiences with a random sampling protocol. Applying random sampling with a sufficient daily frequency allows for time budgeting—enabling statements about daily patterns—and avoids sampling the same situations/environments every day. These types of studies would already help advance restoration research (see, e.g., [51]), but combining it with ambulatory assessment of the context would be even more beneficial.

To illustrate the opportunities, we presented earlier studies looking into the vitalizing effects of exposure to natural environments [50,51]. The use of a similar design on a larger sample would allow for disentangling the temporal dynamics of nature, mood, and preference. For example, time-structured analyses could investigate whether persons go out into nature because they feel alive and happy, whether persons who (happen to) visit more natural scenes develop better mood and vitality, or whether these processes are in fact bidirectional and strengthen each other.

Advanced statistical analyses, such as time series analysis (see, e.g., [26]), differential equation modeling (see, e.g., [80]), or within person factor analysis (see, e.g., [81]), allow for investigating intra-individual patterns. This can be done with both continuous ambulatory data and self-reported experience sampling data. In designing a suitable experience sampling study, it is important to realize that some of these methods require regular measurement intervals and thereby exclude random sampling protocols. In addition, in order to make reliable and sound within-person estimates one will need a sufficient number of data points per participant, thereby generally requiring a longer measurement period with a higher sampling frequency relative to designs investigating inter-individual effects. The exact number of data points necessary will depend both on the specific research question and the data analysis method chosen.

### 6.3. Bridging the Gap between Laboratory Research and Epidemiological Findings

#### 6.3.1. Rationale

As discussed earlier, the current evidence base of restorative environments consists mostly of acute effects (laboratory/field studies) or general longitudinal health effects (epidemiological/archival research). ESM could help with gaining a better understanding of the mid-term benefits of restorative environments (in terms of days, weeks, or months), especially pertaining to effects of nature on stress and, directly or indirectly, on health. In addition, ESM allows for the investigation of more temporally complex, interactional, bidirectional, or even transactional processes, such as between mood, physical activity, and exposure to restorative environments. The strength, direction, and temporal aspects of the relationships between these constructs are as yet unclear and may only be disentangled through longitudinal prospective sampling.

Second, as opposed to investigating the beneficial effects of restorative environments, researchers are now also investigating how constraints of restoration [82] can negatively impact health. As with the nature–stress–health pathway, evidence in this domain is either based on survey research (e.g., [83,84]) or epidemiological findings (e.g., [85]) and could therefore benefit from ESM to link together these outcomes. In essence, laboratory studies into stress-reducing effects of nature have focused on the *reactivity* hypothesis, measuring stress reactivity to a short-lived, acute stressor. However, increasingly the evidence for the detrimental effects of stress points to prolonged (chronic) stress as the main contributor to the pathological effects rather than acute stressors (see e.g., [86,87,88,89]). Prolonged stress is often the result of perseverative cognition [90]. Ruminating about past stressful events and worrying about potential future stressful situations will activate the same stress response as acute stressors, often with a milder intensity but more prolonged in time. Whereas the instantaneous response to a stressor can be quite intense, it is usually of relative short duration. Instead, when cognition about this stressor lingers, the stress response can be extended long after the event occurred and this has proven especially detrimental. The current laboratory experiments (e.g., [7,77]) have only explored reactivity as a response to an acute stressor. Thus, an important segment within the nature-stress-health pathway—prolonged stress—remains as yet unexplored.

Relatively recently, researchers within the restoration domain have started to look at constrained restoration [82]. It has been postulated that factors both within the individual and within the environment can hinder restoration. For instance, survey research indicated that being professionally active in nature can reduce its restorative potential for both adults [83] and children [84] and that teleworking can reduce the restorative potential of the home [82]. Similarly, in an epidemiological study it was postulated that bad weather conditions can limit the ability to access natural areas and thereby increase depression levels [85]. As these constraints depend both on the individual and on the situation, self-tracking in everyday life could substantially contribute to understanding which constraints matter, for whom, and when.

#### 6.3.2. Self-Tracking Opportunities

As rumination and worrying are an inherent part of everyday life, it makes sense to explore these effects using ESM. This could be achieved by tracking rumination and stress levels in parallel with contextual factors. Adding ambulatory assessment of physiological indices (e.g., heart rate variability) would be highly recommended, especially since prolonged stress can also occur unconsciously [91]. In addition, tracking time–location data on a longitudinal scale allows for incorporating place effects extending beyond the current place of residence or work environment. Debatably, due to mutual dependencies, unraveling people effects from place effects will be hard, if not impossible [64,68], but streams of time-bound EMA data can help demystify temporal dynamics and causal processes. ESM thus provides us with tools to help gain a better understanding of these effects.

Employing relatively short-term (1 or 2 weeks) studies linking prolonged stress with restorative environments will be a first step in linking laboratory findings on stress with epidemiological outcomes on health, but will certainly not suffice. To achieve this, there is also a need for studies monitoring longer-term relations between time-location data and stress and health. This will require a different type of experimental protocol, where special attention should be paid to keeping participants motivated and avoiding drop-out. A commercially available app, such as that introduced by MacKerron and Mourato [53], that gives users control over its content and feeds information back to the user might be a good option. In addition, as more continuous and unobtrusive ambulatory assessment through mobile sensing can be employed and less input by users themselves is required, it will become easier to keep users motivated to continue their participation. In other words, this type of research will require a long-term commitment of both participants and researcher and the research team will probably need to resort to what we labeled “next-generation” ESM protocols.

To fully understand the exact nature and effects of constraints on restoration, one will have to look beyond retrospective and epidemiological data. As a first exploration, building a large database containing a rich collection of contextual factors (e.g., who people are with, what they are doing in the environment, weather data) and restoration outcomes (both self-reported and physiological), combined with data mining techniques could help establish relevant constraints. In addition, this area of research would also benefit from testing these constraints in longitudinal protocols.

### 6.4. Advancing Theoretical Insights by Measuring Effects in Everyday Life

#### 6.4.1. Rationale

More detailed investigation of the proposed underlying mechanisms (*i.e.*, resource depletion and stress reduction) constitutes the fourth and last main area of research that we hypothesize could benefit highly from ESM. Self-tracking methods add to laboratory findings by allowing the researcher to sample additional manifestations of stress and self-regulation, such as perseverative cognition and daily temptations. These phenomena are strongly intertwined with the daily hassles and challenges we face and are difficult to replicate in laboratory settings. Investigating the salutogenic effects of the environment on resilience as well as resource capacity therefore seems a logical next step in the quest toward the study of restorative environments.

Besides the stress-reducing potential of nature, evidence points to its ability to overcome resource depletion (also labeled attention fatigue), as described in Attention Restoration Theory (ART; [9]). Extending on this assertion, it has recently been suggested that self-regulatory capacity as described in Ego-Depletion Theory [92] could significantly benefit from exposure to nature [10]. Ego-depletion research currently effectively combines controlled laboratory research (for an overview, see: [93]) with experience sampling of everyday temptations [94,95].

To date, a limited empirical evidence base exists for the beneficial effects of natural environments on self-regulation [77,96]. Similar to prolonged stress, self-regulation is an inherent part of our everyday lives as it entails a wide variety of behaviors including impulse control, resisting temptations, controlling emotions, volition, and cognitive performance [92,93]. In addition, just as the resource for directed attention is postulated to fluctuate throughout the day in response to the exertion of it [9], self-regulatory capacity has also been claimed to be limited [92]. Tracking self-regulatory performance in its many shapes and forms in everyday life would therefore be a suitable candidate for advancing our understanding of the link between restorative environments and self-regulatory capacity, as it captures self-control in daily life but is also sensitive to fluctuations in capacity.

Investigating restorative effects in everyday life also enables us to assess effects on possible restorative outcomes that have previously received little attention, such as sleep quality or success in maintaining interpersonal relations.

#### 6.4.2. Self-Tracking Opportunities

Opportunities for investigating (prolonged) stress in everyday life have already been discussed in the previous section. Similarly, many possibilities exist for testing self-regulation capacity in daily life. ESM protocols could range from self-reported self-regulatory capacity (e.g., ability to concentrate or lapses in temptations) to repeated administration of self-regulatory tasks (e.g., performance on a vigilance task or testing endurance on the handgrip task) in everyday life. Besides enriching the ESM protocol with contextual sensing, physiological monitoring is also of importance as, for instance, Heart Rate Variability has been linked with self-regulatory capacity [97].

Mobile and wearable sensing equipment further enables testing new restoration outcomes, such as sleep quality, measured with an Actiwatch**^®^** or unobtrusive sensors installed in the bed of the participants (e.g., Beddit sleep tracker**^®^**). Provided that the sensors are validated, these outcomes could substantially aid in understanding the relationship between exposure to restorative environments and long-term health benefits.

Besides quantitative research, qualitative assessment of everyday encounters with restorative environments can produce rich information, possibly leading to new theoretical insights. The options here are also legion: using video/audio recordings to categorize the context, using blogs or Vlogs to capture experiences with different environments, asking for verbal responses to questions (and possibly even use social signal processing to analyze the speech component), or using GPS tagging together with short descriptions to gather experiences at favorite or disliked places.

As some psychological phenomena only occur within everyday life and are difficult to replicate in an experimental setting (e.g., temptations), employing (quasi-) experimental setups within the realm of everyday life may also prove very informative.

### 6.5. Quasi-Experimental Studies Using Ecological Momentary Interventions

Rather than trying to artificially induce everyday hassles, temptations, or challenges in the laboratory, one can also try to introduce interventions in everyday life. Ecological Momentary Interventions allows for this, using a (quasi-) experimental setup. Carefully designed interventions could consist of offering participants restorative content on a smartphone or tablet, while at the same time assessing mood and rumination using ESM. Interactive media content is an often-used strategy within mHealth interventions to lower stress or anxiety [30]. For instance, a combination of visual content displaying a virtual island combined with narratives was successful in lowering stress levels among commuting students [98]. The merits of using EMI in restoration research are twofold: it allows for assessing temporal—*i.e.*, quasi-causational—processing and enables measuring effects in the realm of everyday life. This will provide new insights into fluctuations in the efficacy of restorative content as well as the mitigating effects on naturally occurring (prolonged) stress (or other psychological constructs of interest such as self-regulation or mood).

Implementing Ecological Momentary Interventions can go well beyond exposure to natural content. As mentioned in earlier sections, other modalities and environmental characteristics are also of particular interest. Not only can mobile technology be used to show visual content, it can also play sound or direct participants to go to a specific location. This could constitute the advice to sit closer to a window for more daylight exposure, or suggestions to visit a specific location such as a favorite place, the city park, a museum, or the cinema.

Mobile technology can also be used to implement changes in the (smart) environment such as ambient lighting characteristics. Commercial systems (such as Hue**^®^** by Philips) now enable changing light settings (intensity, color temperature) with the single press of a button in an app. Other environmental characteristics, such as indoor temperature or the use of automated blinds in the office, can also be monitored and manipulated using mobile technology. One could test for both static and dynamic protocols (e.g., light exposure that follows the sun in composition), but also interactive patterns (e.g., responding to emotional state of the user or responding to the context such as the amount of daylight available). An additional advantage of this strategy is that it also allows for investigating the relatively “long-term” effects of interventions (at least longer than the short exposure in the laboratory) as well as the cumulative effects and individual preferences of users.

Experimental control over the exact environments a person is exposed to may be achieved by creating location-based triggers. Using GPS coordinates, it is possible to base the sampling strategy on distinct areas or locations. For instance, alarms could sound every time a person enters his or her favorite place. Alternatively, when supplying an information app to visitors of natural areas, one could include a short questionnaire for dedicated areas. These questionnaires could, for instance, probe location-based accounts of wellbeing indices or preference ratings.

Lastly, ESM also provides us with a tool to assess restorative outcomes *after* a design effort has been undertaken to transform a room or building into a healing environment by tracking human behavior, physiology, and cognition before and after the intervention. This could, for instance, be especially viable in clinical settings such as a hospital.

## 7. Conclusions

The range in possible applications of experience sampling in restoration research is as diverse as the seemingly endless possibilities of context-enriched measurement. These vast opportunities are now only gradually starting to be explored, which is also reflected in the fact that many studies report the feasibility of an experience sampling method [35,42,54] rather than the actual outcomes of studies using it.

We have discussed four main areas of restoration research that could benefit from self-tracking, combined with ambulatory assessment of the context. Importantly, even though these areas were presented as being distinct from each other, it is likely that well-designed experience sampling protocols or ecological momentary interventions will advance restoration research simultaneously in multiple areas.

Besides the many strengths of ESM, weaknesses such as reactivity, lower experimental control, and increased self-monitoring should not be overlooked (see, e.g., [20]). In addition, context-enriched and context-sensitive measurements, especially, will demand a multidisciplinary preparation phase, which is also likely to require a substantial time (and monetary) investment. However, the rich dataset collected could be well worth these investments in the end.

Self-tracking offers many research opportunities, but should not be considered the Holy Grail of all future research. The best empirical findings will still originate from a multi-method approach, in which laboratory research and field methods are combined to triangulate theoretically sound evidence. The present paper further stresses the merits of the quantified self movement for research purposes. Paradoxically, however, it could also be argued that extensive self-tracking could contribute to a substantial rise in the—already high—informational burdens of modern life and may therefore contribute to resource depletion.

To conclude, EMA and ESM promise to offer important insights into restorative environments and the underlying mechanisms of recovery. At last, researchers have the tools to put aside persistent criticism and look beyond the strict dichotomy of natural *vs.* urban environments. These methods allow for capturing the salutogenic effects of the environment with a high spatial and temporal resolution as well as expanding the typology of effects under study by incorporating daily hassles and challenges as well as (micro-) restorative [99] experiences, rather than trying to experimentally induce mental fatigue and stress responses in a laboratory environment. These insights will help establish a better understanding of how we can utilize our physical environment to foster and maintain long-term human health and wellbeing.
